# Tumour Movement in Proton Therapy: Solutions and Remaining Questions: A Review

**DOI:** 10.3390/cancers7030829

**Published:** 2015-06-29

**Authors:** Dirk De Ruysscher, Edmond Sterpin, Karin Haustermans, Tom Depuydt

**Affiliations:** 1 Department of Oncology, Experimental Radiation Oncology, KU Leuven-University of Leuven, B-3000 Leuven, Belgium; E-Mails: karin.haustermans@uzleuven.be (K.H.); tom.depuydt@uzleuven.be (T.D.); 2 Center of Molecular Imaging, Radiotherapy and Oncology, Institut de Recherche Expérimentale et Clinique, Université Catholique de Louvain, B-1200 Brussels, Belgium; E-Mail: edmond.sterpin@uclouvain.be

**Keywords:** tumour movement, proton therapy, adaptive radiotherapy

## Abstract

Movement of tumours, mostly by respiration, has been a major problem for treating lung cancer, liver tumours and other locations in the abdomen and thorax. Organ motion is indeed one component of geometrical uncertainties that includes delineation and target definition uncertainties, microscopic disease and setup errors. At present, minimising motion seems to be the easiest to implement in clinical practice. If combined with adaptive approaches to correct for gradual anatomical variations, it may be a practical strategy. Other approaches such as repainting and tracking could increase the accuracy of proton therapy delivery, but advanced 4D solutions are needed. Moreover, there is a need to perform clinical studies to investigate which approach is the best in a given clinical situation. The good news is that existing and emerging technology and treatment planning systems as will without doubt lead in the forthcoming future to practical solutions to tackle intra-fraction motion in proton therapy. These developments may also improve motion management in photon therapy as well.

## 1. Introduction

Movement of tumours, mostly by respiration, has been a major problem for treating lung cancer, liver tumours and other locations in the abdomen and thorax. Organ motion is indeed one component of geometrical uncertainties that includes delineation and target definition uncertainties, microscopic disease and setup errors [1]. Respiratory movements are dependent to some extend on the location of the tumour (e.g., close or away from the diaphragm) and some characteristics of the lungs such as the presence of bullous emphysema [2,3,4,5,6,7]. In case of lung cancer with mediastinal lymph node involvement, it has been demonstrated that the movement of the primary tumour and the lymph nodes is not necessarily in the same phase [7], with the consequence that solutions that only take care of the movement of the primary tumour risk to induce systematic errors for the lymph nodes. The differential movement of the primary tumour and the lymph nodes is not only occurring during the delivery of a radiation fraction, but also between fractions [8]. Besides baseline shifts of primary tumours, the relative position of the primary tumour to individual lymph nodes, between lymph nodes and between the primary tumour, the lymph nodes and the bony anatomy and the carina changes between fractions [9]. It is clear that the optimal coverage of the CTV (clinical target volume) of the primary tumour and of the lymph nodes poses considerable challenges in view of the intra- and inter-fractional movements that are not the same for all targets. Excellent reviews have already been published on tumour movements in proton therapy (for example, [10,11,12,13,14,15]), but here we will place more emphasis on tracking and robust planning.

## 2. Tackling Movements in Photon Therapy

### 2.1. Respiratory Correlated Imaging

At present, four-dimensional (4D) CT imaging has been widely introduced to get insight in the respiration-induced changes and is now considered standard of care in lung cancer radiotherapy [16]. Motion compensated cone beam CT (MC-CBCT) have been developed as well [17]. Because both 4D-CT and 4D-MC-CBCTscans contain artefacts due to residual motion and breathing irregularities, the mid-ventilation and later the mid-position reconstruction scan was developed [18,19]. These methodologies ensure that no systematic errors of respiratory motion are entered into the treatment planning process.

### 2.2. Population-Based Margins

A very frequently used method to take into account geometrical uncertainties are population-based margins [20]. Amongst others, the so-called “van Herk recipe” calculates the margin around the CTV that is needed to deliver at least 95% of the prescribed dose to 90% of the patients [20]. To calculate the CTV to PTV (planning target volume) margin, apart from the SD (standard deviation) of the systematic and random errors, the width of the penumbra modelled by a cumulative Gaussian and the inverse cumulative standard-normal distribution at the prescribed PTV minimum dose level. In the lung where in photon therapy the increased range of secondary electrons results in a broadening of the beam penumbra, the additional margin for random errors is small. Systematic errors therefore have a dominant effect on the cumulative dose, especially in the lungs.

### 2.3. Individualised Margins

The ITV (internal target volume) encompasses all motion and shape changes over the respiratory cycle [21]. In the mid-position (MidP) technique, the time-average position of the tumour and the standard deviation of the motion are derived from the 4DCT scan [20]. The MidP concept leads to smaller volumes than the ITV, without jeopardizing target coverage [22,23]. This is because respiratory motion blurs the dose distribution similar to random errors, indicating that the effect of respiratory motion during treatment delivery is small even for considerable tumour motion. It is clear that this situation is completely different in proton therapy.

## 3. Tackling Movements in Proton Therapy

The interest in proton therapy is mostly fuelled by the dose reduction proximal to the tumour and the absence of dose distal from the target. This results in a decrease of the integral dose to the patient and improved sparing of OAR a few centimetres from the target. The lateral penumbra of protons though is larger than that of photons [24]. Because of the range uncertainties, the distal fall-off of protons is rarely used to spare an OAR that is within 1–2 cm to the target volume in the direction of the beam. Moreover, a simple concept such as the PTV is not suitable for proton therapy as geometrical uncertainties distort the dose distributions because of range uncertainties [25].

In passive systems (broad beams), only range uncertainties caused by anatomy variations need to be addressed. In pencil beam scanning (PBS) systems, however, there is the potential interference between beam delivery dynamics (active delivery) and motion of target and organs at risk. The robustness of the dose distributions will also depend on the type of scanning chosen, uniform scanning or intensity-modulated proton therapy (IMPT). In uniform scanning, every energy layer is scanned uniformly, leading to a flat dose plateau in a homogeneous medium. Heterogeneous anatomies need to be compensated by a range compensator, as in passive systems. In IMPT, there is 3D modulation of the intensity in order to achieve a uniform dose to the target without the need of a range compensator. Every incident beam may deliver a uniform dose (single field uniform dose or SFUD) or only the combination of all incident beams lead to a homogeneous dose. In this review, we will cover active systems only, assuming that all issues related to breathing for passive systems must be addressed for active systems, the reverse statement being not always true.

The effects of motion on proton beam dose distribution has been reviewed elsewhere [10]. Motion affects dose distribution by dose blurring, dose deformation due to anatomy variation and the interplay effect. Possibilities to tackle these problems are margins [11,26,27,28,29,30], minimise motion [31], rescanning [27,32,33,34,35,36,37,38] tracking [39,40,41,42,43,44,45,46,47,48,49,50,51,52,53] and robust planning [54,55,56]. The latter two will be discussed in more detail.

Given the sensitivity of PBS beam spot range calculation based on imaging data, the image quality in terms of motion artefacts will need to be improved. In PBS proton therapy, the total dose distribution also consists of a large number of proton beam spots, showing approximately Gaussian distributions laterally with σ = 4–7 mm, and a typical low dose plateau followed by a sharp Bragg peak (BP) at the distal part of the dose deposition. Any relative displacement of these sharp spot dose depositions will cause local over and under-dosages inside the target volume, hampering the robustness of the dose distributions to motion-related range variations. On the other hand the need for adequate 4D imaging might be higher for proton therapy that for photon therapy.

### Tracking and Gating

Like in photon therapy, beam tracking involves the 3D position of each pencil beam that is adjusted to the real-time variation in patient geometry [39,40,41]. Theoretically, this tackles all the problems of movement. Apart from being technically very demanding in proton therapy, tracking may not be suitable for all moving targets such as mediastinal lymph nodes unless more convenient tracers are developed and the problem of different movement of the primary tumour and each individual lymph nodes can be addressed. However, for some locations such as liver tumours, tracking may be the optimal solution.

In coin lesions in the lung, it may be necessary to implant fiducial markers to visualize the tumour location in planar kV imaging [42]. In general, it is important that the fiducial marker is visible/detectable in the radiographs however for PT one should also consider the perturbations in the dose distribution caused by the implant. Newhauser *et al.* [43] and Giebeler *el al.* [44] have performed Monte Carlo simulations for pelvic PT to investigate the use of different materials and marker sizes in combination with PT, They conclude that stainless steel is more suitable in terms of perturbation then the existing gold markers generally used in photon therapy. Also tantalum has been used in PT of the eye as fiducial marker material. The interest of treating moving tumours with PT will stimulate development for novel types of fiducials in the coming years. A remaining disadvantage of fiducial markers in or close to the target is the limited information about the position of the OAR in the proximity of the tumour. Dedicated study focusing on this issue and the need for that information in PT of moving tumours is required. Also the development of integrated real-time 3D imaging, such as MRI [45], integrated in radiation therapy treatment units currently happening for photon therapy will at some point move to PT.

An interesting technology is prompt gamma [PG] range verification [46], which allows for real-time verification of the BP position during PT delivery. During their transport in the patient, some protons suffer nuclear interactions, leaving the target nucleon in an excited state. The fast component (“prompt”) of the decay process leads to the generation of several secondary particles, including protons, deuterons, alphas and gammas. These gammas may leave the patients and can be imaged by appropriate camera designs [47]. It has been shown that the fall-of of the prompt gamma signal is well correlated with the BP position. Bom *et al.* [48] have investigated a prompt gamma solution, where showed that under common therapy conditions enough data may be collected during one spot-step (in the order of 10 ms). With this they anticipate a performance with this system which could be used to monitor real-time the location of the BP. The question is whether real-time feedback to the BP steering with PG is a possibility. The PG imaging as such delivers information about the range, but no does not contain anatomical information about the target location. When a deviation is detected of range, the only way to respond would be to change the beam energy, to correct for that. The reason why the BP can me multiple, the change of patient position of internal anatomical changes. If however the internal anatomy shifts without large changes in local densities, the PG as such would not deliver any info of even detect a deviation. However, at least PG can become an interesting technology when it comes down to real-time verification any type of respiratory correlated treatment approach.

Once the target volume can be localized in real-time during breathing, it has to be decided what kind of adaptation of the treatment parameters is used to correct for organ motion. Schätti *et al.* [49] advised motion management applying gating, breath-hold or tracking for motion amplitudes exceeding 10 mm. Gating is one approach where the beam on/off is triggered by the target position, whether or not combined with patient involving breath-hold. As was pointed out by Matsuura *et al.* [50], on the short term gated PT approach seems to be the most feasible for use in a clinical setting for compensation of respiratory motion in the near future. Most state-of-the-art PT systems have a beam control interface, allowing beam triggering by 3rd party devices such as optical surface tracking, spirometers or tension belts registering breathing motion. As the selected gate is covering a substantial part of the breathing motion, and there exists inter-cycle variability of breathing motion, it should be further investigated whether rescanning/repainting is required for this level of residual motion in PT. Tsunashima *et al.* [51] have investigated gated PT using a synchrotron-based pulsed beam and described the synchronization issue of the respiratory gate level and synchrotron magnet excitation cycles. For a similar setup Matsuura *et al.* [50] investigated the relation between the dose errors and the motion characteristics, varying different parameters such as BP sharpness, spot size, spot spacing and number of required re-paintings. They also quantified the interaction between the direction of the motion and the spot scanning directions, in terms of dose distortion. Cyclotron based systems with continuous beams or pulsed beam at high frequencies do not suffer from synchronization issues during gated PT delivery. The delay or latency to trigger on/off the beam is of the order of size of a pulse length, hence milliseconds.

For tumour tracking in photon therapy usually the beam aperture is adjusted to the continuously changing tumour position. Solutions such as robotic gantry tracking [52], DMLC tracking [53] and gimballed linac tracking [54] are being used in clinical practice. None of these solutions is taking into account the changes in radiological path length to the target depth, related to changes in local anatomy in the beam portal. As these approaches are usually applied in SBRT settings, it is assumed that this effect is compensated for by the large number of beam orientations. In a PT PBS treatment, where the number of beams usually does not exceed 3, this assumption might not hold.

Essentially the scanning beam of a PBS system could be ideally suited for real-time pursuit of a moving tumour. The scanning magnet speed for lateral deflection of the beam is high enough to adapt the beam spot position to the target position, superimposing motion compensation on the layer beam spot scanning. The location of the pencil beam can be changed every 10 ms. With axial beams in the thoracic region, and the most prominent motion component in craniocaudal direction, this can be handled by the scanning magnets. The question rises whether also energy switching should play a role to compensate for changes in radiological path length. The time resolution is 2 orders of size larger in this depth direction, ranging from 100 ms with mechanical range shifting to 1 s for modification of the energy upstream in the proton beam line. These will result in an internal latency of the system which might have to be compensated by forward prediction of the tumour motion.

## 4. Robust Planning

A plan is said to be “robust” if the treatment plan quality is within requirements in the presence of uncertainties (beam and patient model) [10]. The most satisfactory solution is to explicitly account for these uncertainties in the optimisation cost function. Several methods have been proposed for robust planning [55,56,57]. They minimize either the worst-case dose in every voxel (correlation between voxels disregarded) or the worst-case scenario (correlation preserved). Typically, robust optimisers take as parameters a generic range uncertainty and the magnitude of systematic setup errors in 6 directions. To date, none of the proposed optimisation methods include full 4D optimization, that is, optimizing the dose distributions using all phases of a 4D-CT as already implemented by some groups for photon techniques. Therefore, margins (ITV) are still required to ensure robustness against breathing motion.

The dose calculation algorithm has also a significant influence on the robustness of the treatment plan. Because of the presence of heterogeneities, range uncertainties may be larger with conventional (analytical) algorithms than with accurate Monte Carlo simulations [58]. Dose calculations algorithms based on Monte Carlo simulations are typically much slower than analytical algorithms, which prevented their generalized introduction in treatment practice. However, the simplification of the physics and the introduction of dedicated computing architecture gave rise to Monte Carlo based algorithms able to compute dose distributions in a fraction of a minute [59,60].

The interplay effect may be addressed during robust treatment planning. The effect of breathing motion alone (without interplay) can be quantified by computing the dose from the full treatment plan (all spots) on every data set of a 4D-CT scan. The effect of the correlation between beam delivery and breathing should also be simulated by associating the spots to the breathing phases according to their respective time patterns. Obviously, the resulting dose distribution depends on the initial breathing phase. The contribution of the interplay effect to the degradation of treatment plan quality can then be isolated by analysing the differences between the plan computed with breathing motion alone and the plan simulating the correlation of breathing and delivery motions [61]. Such dose computing scheme could be introduced in a robust optimization loop, which could lead to a pattern of spots that minimizes the interplay effect. Monte Carlo simulations have also here an advantage because their computation time does not scale with the number of CT data sets [62].

## 5. Conclusions

Motion management or intra-fractional movement is being addressed in many studies, but no clinical standard for proton therapy has to the best of our knowledge emerged. The issue is indeed much more complex for proton therapy than for photons. At present, minimising motion seems to be the easiest to implement in clinical practice. If combined with adaptive approaches to correct for gradual anatomical variations, it may be a practical strategy. Other approaches such as repainting and tracking could increase the accuracy of proton therapy delivery, but advanced 4D solutions are needed. Moreover, there is a need to perform clinical studies to investigate which approach is the best in a given clinical situation.

The good news is that existing and emerging technology and treatment planning systems as will without doubt lead in the forthcoming future to practical solutions to tackle intra-fraction motion in proton therapy. These developments may also improve motion management in photon therapy as well.
